# RIPK1 in necroptosis and recent progress in related pharmaceutics

**DOI:** 10.3389/fimmu.2025.1480027

**Published:** 2025-02-11

**Authors:** Kunhou Yao, Zhihao Shi, Fengya Zhao, Cong Tan, Yixin Zhang, Hao Fan, Yingzhe Wang, Xingwang Li, Jun Kong, Qun Wang, Dingxi Li

**Affiliations:** ^1^ Department of General Surgery, Huaihe Hospital of Henan University, Kaifeng, China; ^2^ School of Basic Medicine, Henan University, Kaifeng, China; ^3^ Department of Gynaecology, The Affiliated Cancer Hospital of Zhengzhou University & Henan Cancer Hospital, Zhengzhou, China

**Keywords:** RIPK1, necroptosis, programmed cell death, inflammation, kinase domain, pharmaceutics

## Abstract

Necroptosis is a programmed form of cell death. Receptor-interacting serine/threonine protein kinase l (RIPK1) is a crucial protein kinase that regulates the necroptosis pathway. Increased expression of death receptor family ligands such as tumor necrosis factor (TNF) increases the susceptibility of cells to apoptosis and necroptosis. RIPK1, RIPK3, and mixed-lineage kinase-like domain (MLKL) proteins mediate necrosis. RIPK1-mediated necroptosis further promotes cell death and inflammation in the pathogenesis of liver injury, skin diseases, and neurodegenerative diseases. The N-terminal kinase domain of RIPK1 is significant in the induction of cell death and can be used as a vital drug target for inhibitors. In this paper, we outline the pathways of necroptosis and the role RIPK1 plays in them and suggest that targeting RIPK1 in therapy may help to inhibit multiple cell death pathways.

## Highlights

- RIPK1 is a crucial protein kinase regulating necroptosis
- Increased expression of death receptor ligands enhances susceptibility to necroptosis
- Targeting RIPK1 may inhibit multiple cell death pathways.

## 1 Introduction

Necroptosis is a regulated necrosis mediated by several death receptors. This form of necrosis works against pathogen-mediated infections, morphologically characterized by cell swelling followed by rupturing of plasma membrane (1). It can be initiated by the interaction between ligands and intracellular and extracellular receptors, such as TNF receptor, Toll-like receptor, ZBP1/DAI. The involvement of caspase-8 is a key determinant in the choice between apoptosis and necroptosis (2). RIPK1 serves as the central player in cell death signal transduction, overseeing the activation of NF-κB pathway, apoptosis and necroptosis. Of the various cell necrosis mechanisms, the pathway mediated by TNF/TNFR1 has garnered the most attention from research entities and institutions. It is important to note that TNF-induced necroptosis can take place both in presence and absence of Caspase-8 inhibition (3).

Upon the binding of TNF and TNFR1, TRADD facilitates the assembly of RIPK1, E3 ubiquitin ligase, TRAF 2, TRAF5, cIAP1, and cIAP2, forming what is termed complex I. In subsequent processes, RIPK1 collaborates with RIPK3 through the RIP isoform interaction motif, resulting in a necrotic complex. RIPK3, after autophosphorylation activation, recruits MLKL. This leads to MLKL undergoing conformational changes due to RIPK3-mediated phosphorylation. Such changes resulted in the exposure of the 4HB domain, thereby activating MLKL. Once activated, MLKL assembles into oligomers, involving into higher-order aggregates that travel to the cell membrane and culminate in hot spots. When these hot spots surpass a certain threshold, the cell membrane is compromised, culminating in cell necroptosis (3, 4).

The regulation of this pathway involves numerous components. Necroptosis induced by exogenous pathological agents, such as viral RNA or DNA, can be mediated without the participation of RIPK1. Proteins containing RHIM, such as TRIF or ZBP1, can also collaborate with RIPK3 to initiate necroptosis (5). The core mechanism of necroptosis involves RIPK3 and MLKL. The recruitment of RIPK1 in necroptosis is contingent upon the specific signal receptor activated (6, 7).

## 2 Molecular structure of RIPK1

RIPK1, known as receptor-interacting serine/threonine protein kinase l, is an essential protein characterized by its dual kinase-dependent and scaffolding functions. It has a molecular weight of approximately 76 kDa. Structurally, RIPK1 comprises three primary domains: an N-terminal serine/threonine kinase domain, an intermediate domain, and a C-terminal death domain (DD).

Notably, the intermediate domain houses the receptor-interacting protein (RIP) isoform homotypic interaction motif, known as RHIM (8). Studies have highlighted that each component of RIPK1 has distinct functional roles. Specifically, the N-terminal serine/threonine kinase domain plays a pivotal role in inducing cell death and holds potential as a target for therapeutic molecules, particularly those aimed at small molecular inhibitors (9).

The intermediate structure domain of RIPK1 plays a crucial role in the activation of NF-κB and supports RHIM-dependent signaling. The presence of the RHIM domain facilitates the interaction of ripk1 with other proteins that also contain RHIM. Some of these interacting partners are RIPK3, TRIF, ZBP1 (which is also referred to as DAI or DLMI), and the M45 protein from mouse cytomegalovirus (MCMV) that contains TIR domains.

RIPK1’s C-terminal part is characterized by a death domain (DD). This domain has a multiple role. On the one hand, when presented with matching homologous ligands, the DD anchors RIPK1 to receptors on the cytoplasmic membrane through DD-mediated homotypic interactions. This association is observable with receptors such as TNFR1, Fas, DR4, and DR5. On the other hand, RIPK1’s DD can bind with proteins that also possess a DD, resulting in the formation of intracellular complexes that propagate downstream signaling pathways. Key partners in these interactions are the FAS-related death domain protein (FADD) and the tumor necrosis factor receptor-1-associated death domain protein (TRADD). Additionally, RIPK1’s ability to form homodimers relies on its DD, a critical step that promotes autophosphorylation of the trans-N-terminal kinase domain, leading to its activation (9).

## 3 The role of RIPK1 in necroptosis

RIPK1 plays a central role in the activation of necroptosis, a prominent non-apoptotic cell death pathway. Alongside this, although activation of autophagy may be associated with necrosis, autophagy activation is a consequence of necrosis and does not mediate necrosis (10). Apoptosis has significant implications in various physiological processes, such as routine cell renewal, immune system development and functioning, among others. Excessive necroptosis can precipitate a range of human diseases, encompassing ischemic injuries, neurodegenerative disorders, autoimmune diseases, and even cancer. Necrotic apoptosis is considered as an immunogenic cell death that can activate anti-tumor immunity in tumor cells, and it also participates in promoting adaptive immune suppression induced by myeloid cells, thereby promoting tumor development (11). Thus, research into necroptosis offers potential therapeutic avenues for these diseases.

The initiation of necrosis can be attributed to diverse extracellular triggers, including members of the TNF superfamily, such as TNF-α and the CD95 receptor/Fas ligand complex. Central to the necroptotic signaling process are molecules like RIPK1, RIPK3, and the mixed lineage kinase domain-like protein (MLKL). These form unique multiprotein assemblies that drive the cell towards death.

Investigating further into the signaling mechanics, the TNF-α model highlights three distinct pathways that give rise to necroptosis-specific protein structures. At first, TRADD is recruited to TNFR1 by DD- binding. Then, in the presence of cIAP1 and cIAP2, ligand binding instigates the cIAP1/2 to couple with the TN receptor 1 (TNFR1). This, in conjunction with the TNF receptor-associated factor (TRAF2) and the receptor-mediated protein kinase, culminates in the formation of Complex I. This complex amplifies the polyubiquitination of RIPK1. However, in the absence of cIAP1/2, complex I disintegrates, causing RIPK1 to detach from the TNFR1. It then forms Complex IIa with the Fas-associating protein with a novel death domain (FADD), typically leading to Caspase-8-dependent apoptosis.

**Figure 1:**
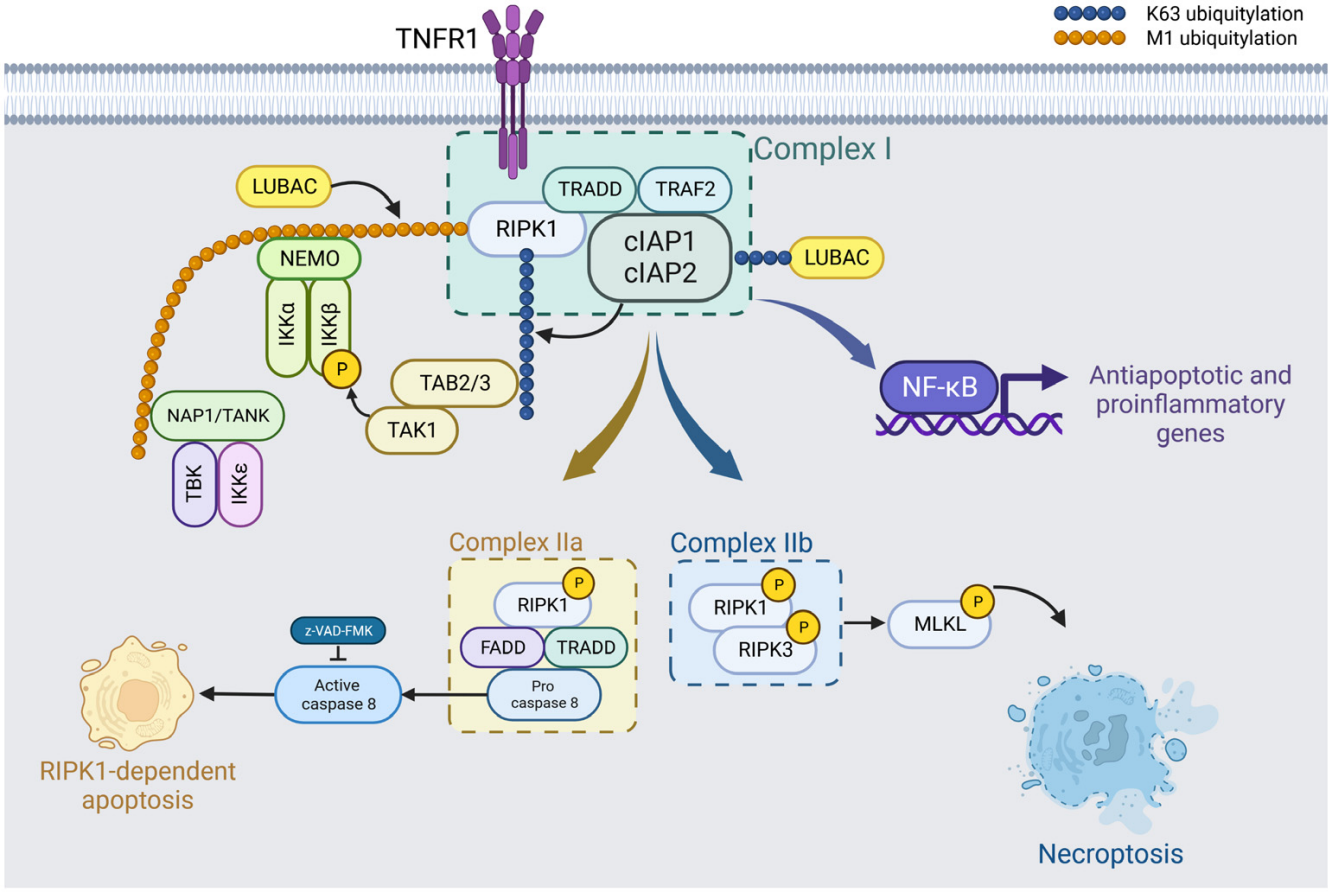
TNF-α-driven formation of necroptosis-specific protein complexes. Upon binding of tumor necrosis factor (TNF-α) to TNF receptor 1 (TNFR1), the presence of cIAP1 and cIAP2 facilitates their association with TRADD, TRAF2, and other receptor-mediated protein kinases to form Complex I. This complex promotes the polyubiquitination of RIPK1, which then activates the NF-κB pathway, leading to the induction of anti-apoptotic and pro-inflammatory genes. Kinases such as IKKα/β/ϵ, TBK1, and TAK1 phosphorylate RIPK1. These phosphorylation events negatively regulate RIPK1 activity, thus inhibiting subsequent cell necrosis. Created in BioRender.com. Shi, Z. (2025) https://BioRender.com/k68l223.

Alternatively, when the action of Caspase-8 is hindered, RIPK1, in tandem with Complex IIa, phosphorylates RIPK3, resulting in the creation of Complex IIb or the necrosome. This necrosome then acts to further phosphorylate MLKL. This phosphorylation prompts a conformational shift in MLKL, relocating it to the plasma membrane. This relocation disrupts cell membrane integrity, ultimately setting the stage for subsequent cell death (9) (See **Figure 1**).

In the scenario where cIAP1 and cIAP2 are absent, Complex I dissociates. RIPK1 then detaches from this complex, pairing with Caspase-8 and FADD (Fas-associated death domain) to form Complex IIa. Cell death resulting from this configuration is termed RIPK1-dependent apoptosis. If Caspase-8 activity is additionally inhibited, phosphorylated RIPK1 and RIPK3 associate to form Complex IIb. Following this, MLKL is recruited, activated, and assembles in a manner that compromises the cell membrane, resulting in cell death. This mechanism is known as RIPK1-dependent necroptosis. Created with BioRender.com.

RIPK1 serves as a vital component in the mediation of both apoptosis and necroptosis, downstream of the death receptor (DR) and pattern recognition receptor (PRR). Moreover, RIPK1 plays an instrumental role in the inflammatory pathway (12). RIPK1 can either acting as a scaffold to shield cells from death or as an active kinase to promote cell death. Within the context of the TNF-α signaling model, RIPK1 has been identified as a critical player in the four branches of the TNFR1 response.

### 3.1 RIPK1 inhibits necroptosis by functioning as a scaffold

RIPK1-mediated ubiquitin-dependent signal transduction is pivotal for the activation of NF-κB, which in turn promotes the transcription of survival genes. One such protein product is cFLIP (Cellular FLICE-like inhibitory protein), which acts to deter apoptosis by interacting with Caspase-8, preventing its activation. During development, the RHIM domain of RIPK1 plays a central role in averting ZBP1/RIPK3/MLKL-dependent necrosis (13). RHIM in RIPK1 prevents ZBP1 from activating RIPK3 upstream of MLKL, since both RIPK1 and ZBP1 contains RHIM that are interactable. Should the RHIM function of RIPK1 be compromised, ZBP1-triggered necrosis ensues.

### 3.2 RIPK1 promotes necroptosis as an active kinase

Necroptosis is orchestrated by the kinase activity of RIPK1, in collaboration with RIPK3 and MLKL. The autophosphorylation at S166 within the RIPK1 kinase domain serves as an indicative marker of RIPK1 activation. Genetic investigations have revealed that the autophosphorylation of RIPK1 at S166 is required for RIPK1 kinase-dependent cellular death and inflammation (14). The kinase activity inherent to RIPK1 is crucial in mediating both cell necroptosis and apoptosis.

### 3.3 TRAIL: a trigger of necroptosis

TNF-related apoptosis-inducing ligand (TRAIL) is a death receptor (DR) ligand that mediates cell apoptosis and necrosis. It establishes a receptor-binding RIPK1-independent death signaling complex, subsequently inducing Caspase-8-dependent apoptosis. When Caspase is inhibited, TRAIL facilitates the recruitment of RIPK1 to Fas (15), resulting in the formation of Ripoptosome (Rip protein apoptosome), which culminates in necrosis.

At a neutral pH of 7.4, with low ATP depletion, TRAIL triggers apoptosis in approximately 30% of cells, excluding neutrophils. However, in an acidic environment with a pH of 6.5, necroptosis occurs in about 70% of cells, causing significant ATP depletion. Characteristically, ATP depletion levels of 80-85% or higher are indicative of necrosis. Nec-1 robustly suppresses TRAIL-induced necrosis. Pretreatment with Nec-1 redirects the cell death pathway from necrosis to apoptosis under acidic conditions and lessens intracellular ATP depletion (16).

Notably, the programmed cell death induced by TRAIL and the effect of Nec-1 in mitigating ATP depletion are mutually inhibitory. Knockout (KO) experiments with Ripk1 affirmed that TRAIL-induced necroptosis was capable of preventing necrotic cell death and exhibited Nec-1 depletion similar to that observed with Nec-1 treatment. In mouse embryonic fibroblasts (MEF) with RIPK1-KO, TRAIL-driven necrosis was suppressed in acidic conditions. This highlights the essential roles of both RIPK1 and RIPK3 in TRAIL-mediated necrosis when cells are exposed to an acidic environment.

## 4 Regulation of RIPK1

**Figure 2:**
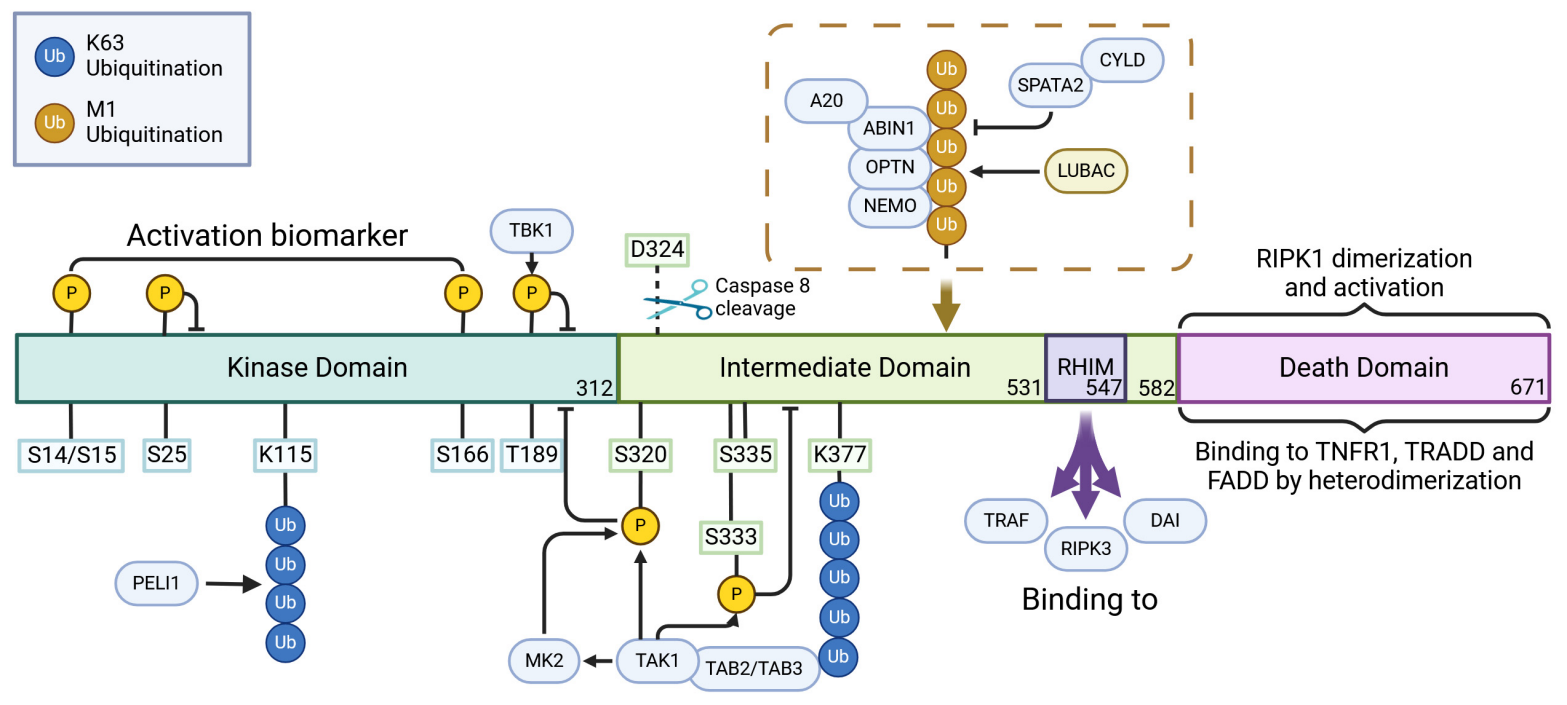
The regulatory role of posttranslational modifications in the activation of RIPK1. RIPK1 possesses a structured hierarchy: an N-terminal kinase domain (KD), an intermediate domain (ID), and a carboxyl-terminal Death Domain (DD). The KD, pivotal for RIPK1’s function, achieves self-activation through self-phosphorylation. Interestingly, Caspase cleavage at D324 severs the connection between KD and DD, effectively thwarting RIPK1 activation. In its activation journey, RIPK1 undergoes self-phosphorylation at sites like S14, S15, and S166. Yet, phosphorylation at particular kinase domain sites, such as S25, S189, S321, and S320 by regulators like IKKs, TBK1, TAK1, and MK2, serves as a countermeasure, inhibiting its kinase activity and stalling necroptosis. The E3 ubiquitin ligase, pellino 1 (PELI1), plays a special role: it ubiquitinates K115 within the KD of an activated RIPK1, which aids in the assembly of complex IIb, thereby promoting necroptosis. Moreover, the interaction of RHIM motifs present within the ID of RIPK1 and RIPK3 acts as a conduit for necroptotic and inflammatory signaling. But, it’s not all pro-necroptosis; the ubiquitination of multiple sites in RIPK1’s ID aids in the transmission of the NF-κB signal, acting as a necroptosis deterrent. Here, K377 stands out, serving as a nexus point for E3 ubiquitin ligases like IAP1, IAP2, and parkin. Once ubiquitinated, K377 becomes an anchor, attracting activators such as TAB1/2 and NEMO of TAK1. This recruitment ultimately leads to a cascade activation of TAK1, IKK, and MK2. These entities, once activated, reciprocate by exerting inhibitory phosphorylation on RIPK1. In the realm of DD, RIPK1 interacts to form homologous dimers with partners like TNFR1, TRADD, and FADD, which act as triggers for KD activation. CYLD, brought into play by the LUBAC, serves dual purposes: it facilitates necrotic body formation by stripping K63 and M1 ubiquitin chains, and paradoxically, impedes the NF-κB signaling, enhancing necroptosis. Meanwhile, A20 is versatile. As a K63 ubiquitinase, it escalates necroptosis by firing up RIPK1 and RIPK3. Yet, it’s also a proficient ubiquitin ligase, marking RIPK1 for proteasomal disposal and thus modulating RIPK1-driven necroptosis. The narrative doesn’t end here; PELI1, OPTN, Parkin, and ABIN-1 each play their parts, influencing the NF-κB signaling pathway and subsequently the fate of RIPK1-mediated cell death. Created in BioRender.com. Shi, Z. (2025) https://BioRender.com/l77i832.

RIPK1’s activity is intricately modulated by a spectrum of post-translational modifications (PTMs), encompassing processes such as ubiquitination, phosphorylation, and the precise cleavage mediated by Caspase-8 (See **Figure 2**).

### 4.1 Phosphorylation

Through rigorous research, we have identified specific phosphorylation sites that influence RIPK1 activity and, consequently, modulate death signal transduction. These pivotal sites encompass autophosphorylation regions that activate RIPK1, propelling cells towards a necrotic fate, as well as phosphorylation sites formed by external kinases that serve to restrain RIPK1 activity (3). The phosphorylation is mainly mediated by TBK1, TAK1, IKK, MK2 (18).

The absence of TBK1 has been demonstrated to heighten the cellular susceptibility to apoptosis. In human RIPK1, T189 is a threonine residue pivotal for substrate recognition. TBK1 modulates RIPK1 activity with T189 acting as a direct substrate. Cells devoid of TBK1 exhibit heightened sensitivity to TNF-α (tumor necrosis factor), suggesting TBK1’s inhibitory role in RIPK1-mediated cell death (19, 20). Suppression of TAK1 hinders the pro-survival NF-κB signaling stimulated by TNF-α and augments RIPK1 phosphorylation (21).

Furthermore, in the absence of TAK1, bone marrow-derived cells undergo spontaneous RIPK1-dependent death (22, 23). These findings underscore TAK1’s role as a repressor of RIPK1 kinase activity (24).

The phosphorylation of RIPK1 at the S25 site by IKKs is instrumental in directly suppressing RIPK1 activity and averting RIPK1-dependent cell death. When IKK activity is suppressed, pseudophosphorylation at S25 (manifested as the S25D mutation) shields cells from TNF-induced cytotoxicity. Notably, in TAK1-deficient cells, TNF-induced RIPK1 S25 phosphorylation is abrogated (25). Experiments on L929 cells (mouse fibroblasts) treated with a TBK1/IKKϵ-specific inhibitor showed heightened cell death upon TNF stimulation. Subsequent tests revealed that both kinases could directly phosphorylate RIPK1, dampening its autophosphorylation (26). NEMO facilitates the recruitment of TBK1 and IKKϵ, suggesting that mutations in NEMO can lead to TBK1 and IKK deficiencies, resulting in aberrant TNF-induced cell death.

In the TNFR1 signaling pathway, MR2 phosphorylates RIPK1 at sites S321/336 (or S320/335 in human RIPK1). This phosphorylation constrains RIPK1 activation via autophosphorylation and inhibits RIPK1’s association with FADD/Caspase-8, thereby curbing RIPK1-mediated cell death (27–29). The function of IKKα/β differs from that of MK2 in phosphorylating RIPK1. Under conditions of IKKα/β inactivation, the suppression of M2 heightens cellular vulnerability to TNF stimulation, leading to elevated cell death rates (30). Several RIPK1 phosphorylation sites dependent on ULK1 were identified, including Ser262, Ser291, Ser296, Ser320, and others. Prior experiments revealed that ULK1 could diminish RIPK1 autophosphorylation, serving as a negative regulator of RIPK1 activity to stabilize complex I. This suggests ULK1’s role in downregulating TNF-mediated cell necroptosis (28).

### 4.2 Autophosphorylation

The autophosphorylation at the S166 site within the RIPK1 kinase domain serves as an indicator of RIPK1 activation. Autophosphorylation at S166, which may induce conformational shifts within the RIPK1 protein, potentially facilitates autophosphorylation of other sites and enhances the accessibility of the RIPK1 kinase domain, thereby promoting interactions with other substrates or regulatory proteins (14). For instance, S166 autophosphorylation may facilitate the interaction between RIPK1 and death domain-containing proteins, including FADD and RIPK3, which are essential for necrosome assembly and the propagation of cell death signaling (31). Some experiments have revealed that the expression level of the Ripk1S166A mutant protein mirrors that of the wild type (WT). This observation suggests that the S166A mutation does not detrimentally affect RIPK1’s role in initiating inflammation and maintaining tissue homeostasis. Subsequent research has shown that the suppression of autophosphorylation at the S166 site does not alter RIPK1’s scaffolding function in modulating the proinflammatory and survival pathways of NF-κB and MAPK. Nevertheless, autophosphorylation of RIPK1 at the S166 site is paramount for necroptosis induction. Nullifying autophosphorylation at this site markedly curtails the kinase-dependent cell death activity of RIPK1 downstream of TNFR1, TLR3, and TLR4 (14).

Furthermore, it has been posited that phosphorylation at the S161 site could enhance the interaction between RIPK1 and RIPK3, facilitating the formation of necroptosis and thereby spurring cell death (32). Given that S161 resides within a potential regulatory loop that interacts with the Necrostatin-1 (Nec-1, an inhibitor of RIPK1) (33), a pertinent question arises: Does the S161 mutation influence RIPK1’s sensitivity to Nec-1? Upon delving into available data, it was noted that the S161A mutation only dampened RIPK1’s ability to mediate TNF-induced necroptosis, without completely nullifying RIPK1 activity (34). When S161 is mutated to the more negatively charged amino acid, Glutamate (Glu), there is a tenfold decrease in the inhibitory efficiency of Nec-1 (35).

### 4.3 Ubiquitination

Within complex I, RIPK1 undergoes various ubiquitination modifications, including M1, K11, K48, and K63 ubiquitination. These modifications are pivotal in modulating RIPK1 activation. Research has shown that specific E3 ligases and ubiquitinases can mediate these ubiquitin modifications, which in turn can either enhance or suppress RIPK1-mediated cell death (17).

### 4.4 Activation of RIPK1 mediated cell death is regulated by various ubiquitinations

The K622 (in humans) or K612 (in mice) site is pivotal for RIPK1 ubiquitination. Ubiquitination at K622 facilitates the interactions between RIPK1 homodimers and RIPK1-TNFR1 complexes, which are mediated through their respective death domains, culminating in the activation of RIPK1 (36). Following TNFα stimulation, cells with RIPK1 that underwent M1 and K63 ubiquitination saw a decrease when subjected to a K612R mutation. This suggests that ubiquitination at the K622/K612 sites can augment RIPK1-mediated cell death, signifying their integral role in the overall ubiquitination regulation of RIPK1 (37).

CYLD is channeled to LUBAC via the SPATA2 adapter. As a deubiquitinating enzyme, CYLD removes K63 and M1-linked ubiquitin chains from RIPK1 and TRAFs, subsequently allowing RIPK1 to associate with RIPK3 and form necrosomes. Moreover, CYLD can dampen NF-κB activation via TNFRs, propelling RIPK1-mediated cell death. Additionally, CYLD has been identified as a deubiquitinase for TAK1. CYLD can thwart both apoptotic and necroptotic signals induced by TAK1 inhibition, suggesting its pivotal role in RIPK1 activation, especially in subsequent necrosome formation under TAK1 inhibition (3, 24, 38, 39).

The E3 ligase Pellino 1 (PELI1) orchestrates K63 ubiquitination on Lys115, activating RIPK1 and fostering the assembly and necroptotic actions of complex IIb. Silencing PELI1 diminishes TNF-α-mediated polyubiquitination of TRAF6 and RIPK1, curbing NF-κB and P38 activation. ChIP assays and luciferase reporting indicate that the binding of NF-κB and AP-1 to TGFβ1 promoter regions is impeded in the absence of PELI1 expression, thereby inhibiting TGFβ1 activation. The absence of PELI1 escalates the number of cells undergoing either RIPK1-dependent or RIPK1-independent apoptosis (38, 40, 41).

The deubiquitinase OTULIN specifically cleaves the linear ubiquitin chains generated by LUBAC. In one study, researchers discerned that OTULIN limits both apoptosis and necroptosis in keratinocytes. During apoptosis, OTULIN is cleaved into a C-terminal fragment at the Asp-31 site by Caspase-3, curtailing Caspase activation and subsequent cell death. Conversely, during necroptosis, OTULIN undergoes hyperphosphorylation at the Tyr56 site, augmenting RIPK1 ubiquitination and promoting cell death. This phosphorylation of OTULIN’s Tyr56 can be counteracted by DUSP14 (42).

### 4.5 Down-regulation of RIPK1 mediated cell death by ubiquitination

OPTN, an ubiquitin-binding protein, reduces K48 ubiquitination, slowing the degradation of RIPK1 and contributing to its stability (43). The K63 polyubiquitin chains on RIPK1 and other components of complex-I function as anchor points for key activating factors in the NF-κB pathway, such as TGF-β, TAK1, TAB1/2, and NEMO, which further stimulate the activation of TAK1 and IKK complexes (44).

Various E3 ligases, including TRAF2/5, cIAP1/2, and LUBAC, can catalyze multiple ubiquitination events on RIPK1. This fosters the recruitment of TAK1 and protein complexes, which include NEMO (NF-κB essential modulator) and IKK α/IKK β, thereby activating the NF-κB signaling pathway and indirectly diminishing cell death (45). It’s been shown that K376 (corresponding to human K377) undergoes ubiquitination by cIAP1/2 (46). This site offers a docking point for the K-63-linked polyubiquitin chain and plays a role in the recruitment of TAK1 and IKK complexes. Experiments demonstrated that replacing lysine-377 with arginine (K377R) prevents RIPK1 ubiquitination and inhibits NF-κB signaling, making cells more susceptible to TNF stimulation. However, this finding is based on *in vitro* results, casting uncertainty on its *in vivo* function (47). The absence of cIAPs impedes the activation of NF-κB, and it also alleviates the MK2 and IKK-mediated inhibition of RIPK1. Therefore, cIAPs ubiquitination at K376 site hinders RIPK1 activity (48).

The linear ubiquitin chain assembly complex (LUBAC) plays a vital role in cell signaling, consisting of key components such as heme-oxidized IRP2 ubiquitin ligase 1 (HOIL-1) and shank-associated RH domain-interacting protein (SHARPIN). HOIL-1-interacting protein (HOIP), the catalytic subunit within LUBAC, exhibits a unique function. HOIP deficiency does not interfere with RIPK1’s recruitment to TNF-RSC. Instead, it promotes RIPK1’s association with complex IIb, a process determined by the interaction between RIPK1 and FADD. The presence of HOIP can mitigate FMK-induced necroptosis and temper RIPK1 activation (42).

Parkin, a specific cytosolic E3 ubiquitin ligase, facilitates K63 ubiquitination on RIPK1 K376, bypassing cIAP1/2. Upon TNF-α exposure, Parkin accelerates the recruitment of various signaling molecules, such as TAK1, NEMO, Sharpin, and A20, to the TNFR1-associated complex I. Following ubiquitination by Parkin, RIPK1 undergoes enhanced phosphorylation of IKK α/β and IκBα, leading to the activation of the NF-κB and MAPKs pathways. This results in the nuclear translocation of p65, promoting cell survival while countering RIPK1-mediated cell death (44).

ABIN-1, a ubiquitin-binding protein, plays a pivotal role in staving off apoptosis, necroptosis, and in activating NF-κB. Its recruitment by the TNFR1 signaling complex enhances the association of A20. A20, acting as a K63 deubiquitinase, curtails the activities of RIPK1 and RIPK3, thus repressing cell necroptosis. The carboxyl-terminal zinc finger region of A20, combined with the amino-terminal OTU motif, endows it with dual enzymatic functions. Besides its deubiquitinase capability, A20 can act as a ubiquitin ligase, targeting RIPK1 for proteasomal degradation (49, 50).

### 4.6 Other pathways related to RIPK1 regulation

Through in-depth investigations, MIB2 has been identified as an E3 ligase that displays the capability to impede cell death via dual mechanisms. Firstly, MIB2 can hinder RIPK1-dependent apoptosis by suppressing the activity of RIPK1. Secondly, it modulates the ubiquitination of cFLIPL, a Caspase-8 analog, thus inhibiting death receptor-mediated apoptosis or what is termed as RIPK1-independent apoptosis (51).

cFLIPL’s interaction with Caspase-8 results in the formation of a heterodimer. This dimerization action obstructs Caspase-8 self-processing, which is a fundamental precursor for cells to undergo RIPK1-independent apoptosis. In such a scenario, RIPK1’s activity is sustained, aiding in the prevention of cell death. Furthermore, Caspase-8 has an additional role in negating necroptosis: it achieves this by enzymatically cleaving RIPK1 at the Asp325 site, which also incapacitates RIPK1 kinase activation (52).

Interestingly, while the absence of MIB2 doesn’t alter the formation of complex I that is prompted by NF-κB activation due to TNF induction, it significantly amplifies the genesis of the cytoplasmic death-induced signaling complex II (51).

## 5 RIPK1 and disease

**Figure 3:**
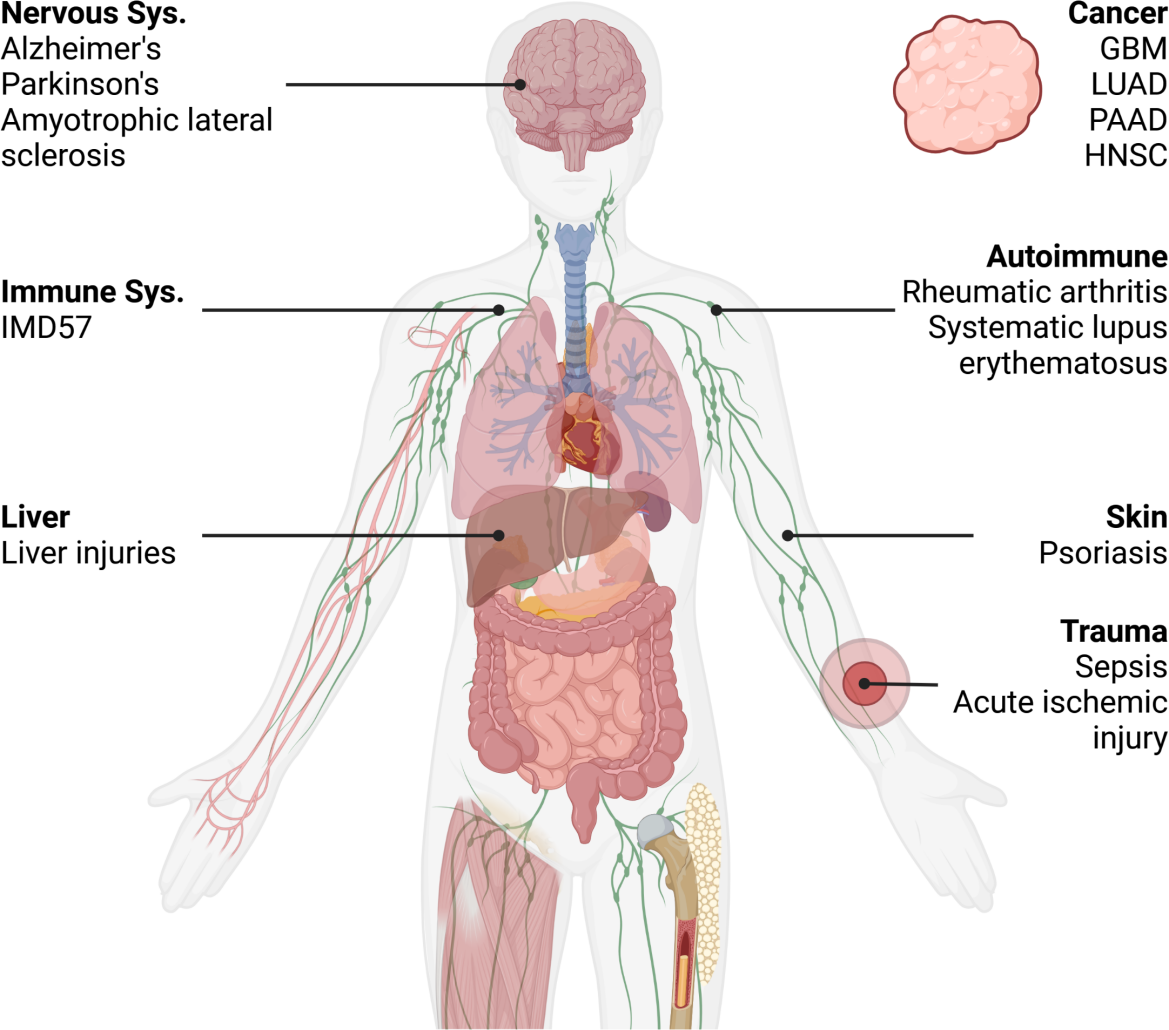
Distribution of RIPK1-related disease mentioned in the article. IMD57, Immunodeficiency 57; GBM, Glioblastoma multiforme; LUAD, Lung adenocarcinoma; HNSC, Head and Neck squamous cell carcinoma; PAAD, Pancreatic adenocarcinoma. Created in BioRender.com. Shi, Z. (2025) https://BioRender.com/o42n771.

Based on our research conducted through the Human Protein Atlas (HPA), we have found that RIPK1 is ubiquitously expressed across various normal organs. NoSupplementary Table Sites of expression include the liver and skin, among others. Numerous studies have extensively explored diseases related to RIPK1 expression, encompassing liver injuries, skin diseases, cancers, and more (See **Figure 3**). In the following section, we will succinctly review the roles played by RIPK1 in these conditions.

### 5.1 RIPK1 and liver injuries

Cell death plays a pivotal role in the progression of liver diseases, and both apoptosis and necroptosis have been identified within the liver. Of the two, apoptosis tends to be the more dominant pathway. Extensive studies have delved into the roles of RIPK1 and RIPK3 across various liver injury models, primarily aiming to discern the role of necroptosis. Hepatocytes prominently express death receptors (DR), and several disease conditions, including Non-Alcoholic Steatohepatitis (NASH), viral hepatitis, alcoholic hepatitis, cholestatic liver disease, and autoimmune liver diseases, have been associated with hepatocyte apoptosis (53, 54).

However, a critical argument raised against hepatic necroptosis is that in many experimental conditions where apoptosis is triggered, averting hepatocyte apoptosis seems to resolve the issue without a shift towards necroptosis (55). The debate remains regarding whether hepatocytes are fully equipped with the necessary machinery for necroptosis.

Attention was drawn to RIPK1 in the context of liver disease due to the discovery of Nec-1, an inhibitor of RIPK1 kinase. *In vitro* studies have shown that Nec-1 can thwart necrotic cell death across multiple organ systems (56–60). Nec-1’s effectiveness in mitigating acetaminophen (APAP)-induced liver injury in mice, as well as enhancing hepatocyte survival *in vitro*, was seen as promising. Given that the dominant cell death pattern in APAP overdose is necrotic, Nec-1’s protective effect was initially interpreted as evidence against necroptosis. However, the specificity of Nec-1 for RIPK1 or necroptosis itself came into question (61). Consequently, there’s a need for more robust evidence to solidify the role of RIPK1 in APAP-induced liver damage (62).

In the context of APAP-induced liver injury, RIPK1 knockdown has been shown to offer substantial protection against cell death, thereby enhancing the survival prospects of mice when using an antisense oligonucleotide knockdown approach. Yet, the role of RIPK1 in APAP-induced liver toxicity remains controversial. Dara et al. (63) highlighted in a murine *in vivo* study that RIPK1’s mediation of APAP liver toxicity is independent of the necrosome and doesn’t proceed through necroptosis. These findings were independently corroborated by other researchers (64). This underscores the idea that the mere involvement or induction of RIPK in liver injury doesn’t necessarily signify necroptotic cell death.

### 5.2 RIPK1 and skin disease

Psoriasis, a chronic skin condition, has drawn attention in the clinical realm, especially with RIPK1 inhibitors being tested in clinical trials as potential therapeutic agents. As such, diving deeper into how RIPK1 modulates the progression of psoriasis is of paramount importance.

In a landmark study, Duan and colleagues employed a mouse model of experimental psoriasis to delve into the necrotic response intrinsic to psoriasis pathogenesis. Their findings revealed significant upregulation of RIPK1 and MLKL in IMQ-induced psoriatic skin in mice, mirroring the same elevation across all epidermal layers observed in human psoriatic lesions. This upregulation was correlated with an augmented necrotic rate. Intriguingly, inhibiting RIPK1 curtailed the RIPK1/RIPK3-mediated necrosis *in vitro*. Through advanced techniques like TUNEL immunofluorescence and immunohistochemical labeling of active Caspase-3, RIPK1, and MLKL, the researchers pinpointed that necrosis predominantly manifested in the upper epidermal layers, especially in keratinocytes—paralleling observations in psoriasis patients (65).

Furthermore, administering the RIPK1 inhibitor, Nec-1s, mitigated the inflammatory response evident in IMQ-induced psoriatic dermatitis mice. The intricate dance between necroptosis and psoriasis became evident; necrotic responses can amplify the psoriatic inflammatory cascade. Simultaneously, the psoriatic inflammatory milieu, by compromising the epidermal barrier, exposes keratinocytes to a spectrum of harmful stimuli, culminating in widespread cellular necroptosis (65).

RIPK1’s multifaceted roles, dictated by its scaffolding and kinase activity, allow it to participate in diverse pathways. Potent inhibitors, targeting RIPK1, have shown promise in preclinical studies. For instance, a notable link between RIPK1 inhibitors and attenuation of melanoma development and metastasis has been documented in mouse models (66).

Clinically, psoriasis treatments often employ necroptosis inhibitors like Nec-1s or NSA. However, the advent of GSK2982772, an orally administered small molecule that inhibits RIPK1 kinase, has changed the game. Boasting superior inhibition and enhanced pharmacokinetic profiles over Nec-1s, GSK2982772 adeptly blocks various TNF-dependent cellular responses by latching onto RIPK1 (65).

Further, clinical findings suggest a possible dose-response relationship, as higher systemic concentrations of GSK2982772 were associated with more pronounced reductions in PLSS scores, hinting at potential dose escalations for better therapeutic outcomes (67).

In sum, the burgeoning research into RIPK1 and its associated pathways is illuminating new regulatory molecules, mechanisms, and innovative inhibitors. These discoveries might herald new therapeutic avenues for skin conditions like psoriasis by specifically targeting necrotic tissues.

### 5.3 RIPK1 mutation and immunodeficiency

RIPK1, as a gene, exhibits significant genetic variability. According to the Residual Variation Intolerance Score (RVIS), approximately 24.5% of mutations in RIPK1 are predicted to be disease-causing. The majority of pathogenic RIPK1 mutations are linked to a specific form of immunodeficiency termed Immunodeficiency 57 (IMD57). Notably, IMD57 presents alongside a myriad of associated conditions such as inflammatory bowel disease, arthritis, recurrent infections, and a lymphocytopenic syndrome.

A seminal clinical study led by Cuchet-Lourenço et al. examined four patients exhibiting a unique compound heterozygous mutation that resulted in total RIPK1 deficiency. These individuals grappled with the debilitating effects of immunodeficiency, manifesting as recurrent infections, early-onset inflammatory bowel disease, and progressive polyarthritis. Genetic sequencing pinpointed mutations in the RIPK1 kinase domains at residues 153, 230, or 289. Functional studies using *in vitro* assays demonstrated that cells devoid of RIPK1 displayed compromised MAPK activation, suboptimal cytokine secretion, and heightened susceptibility to necroptosis. Remarkably, hematopoietic stem cell transplantation administered to one patient ameliorated the cytokine secretion defect and provided noticeable clinical relief.

Corroborating these findings, a study spearheaded by Li et al. shed light on eight patients characterized by RIPK1 deficiency, who were grappling with primary immunodeficiency coupled with intestinal inflammation. In these patients, mutations in RIPK1 correlated with diminished NF-κB activity, perturbations in T- and B-cell differentiation, amplified inflammasome activity, and an impaired response of intestinal epithelial cells to TNFR1-induced cell death.

Zelic et al. investigated the role of RIPK1 kinase activity in TNF-induced systemic inflammatory response syndrome (SIRS), particularly within endothelial cells. They discovered that RIPK1 kinase activity mediates hypothermia and mortality in TNF-induced shock models, indicative of excessive inflammation in SIRS. Notably, the absence of RIPK1 kinase activity did not alter cytokine and chemokine levels but significantly enhanced resistance to TNF-induced shock in mice. Using a RIPK1 kinase-inactive mouse model, the study observed survival rates, cytokine levels, intestinal and vascular permeability, and coagulation cascade activation in TNF-induced SIRS. The conclusion was that RIPK1 kinase activity plays a pivotal role in vascular damage and mortality in TNF-induced SIRS, especially in liver endothelial cells, and its absence protected mice from TNF-induced shock, highlighting a protective effect mediated by non-hematopoietic cells. Clinically, this study suggests that RIPK1 kinase inhibitors may offer benefits for patients with shock and/or sepsis, particularly in preserving vascular integrity and preventing tissue damage (68).

In synthesizing the above insights, it’s clear that RIPK1 wields significant influence over the human immune system’s functions. Consequently, therapeutic interventions targeting RIPK1 might hold substantial promise in ameliorating associated immunodeficiencies.

### 5.4 RIPK1 and cancer

Resistance to apoptosis is a fundamental challenge in cancer therapy. When cancer cells evade apoptosis, it often leads to chemotherapy failure. Circumventing the traditional apoptotic route to induce cancer cell death is an attractive strategy to address this resistance. In this context, necroptosis, an alternative form of programmed cell death, emerges as a potential avenue to bypass apoptosis resistance and may even amplify antitumor immunity during cancer treatment.

Data from the Human Protein Atlas (HPA) and The Cancer Genome Atlas (TCGA) indicate that RIPK1 is expressed across a spectrum of cancers. Notably, its expression varies. In certain malignancies, such as glioblastoma, lung cancer, and pancreatic cancer, RIPK1 expression seems to be augmented. Elevated RIPK1 levels correlate with unfavorable outcomes, suggesting its potential role in promoting tumorigenesis. Conversely, in other cancers, like head and neck tumors, RIPK1 expression appears subdued, yet this downregulation still seems to encourage tumor progression.

These findings suggest a nuanced role for RIPK1 and necroptosis in the context of cancer. While on one hand, they might hinder tumor growth and progression, on the other, they could potentially cultivate an environment conducive to tumorigenesis. This dual nature is reminiscent of a “double-edged sword.” Additionally, the inflammatory response instigated by necroptosis might inadvertently bolster tumorigenesis and metastasis. While there is evidence pointing towards the antimetastatic effects of necroptosis, other studies indicate its potential in promoting tumor growth and cancer spread (69).

### 5.5 RIPK1 and neurodegenerative diseases

Necroptosis plays a pivotal role in neuroinflammation, mediating the pathogenesis of several neurodegenerative diseases, such as multiple sclerosis (MS), amyotrophic lateral sclerosis (ALS), Parkinson’s disease (PD), and Alzheimer’s disease (AD). Studies suggest that targeting RIPK1 might be beneficial in inhibiting various cell death pathways and ameliorating neuroinflammation (70).

Pathological axonal degeneration is commonly observed in patients with neurodegenerative diseases such as ALS, MS, AD, and PD. Oligodendrocyte degeneration and dysfunction stand as central mechanisms of axonal degeneration in these diseases. RIPK1 is crucial in oligodendrocytes’ necroptotic cell death, acting as a vital regulator. Therefore, inhibitors of RIPK1, like Nec-1s, can prevent oligodendrocyte degeneration, offering potential therapeutic avenues for neurodegenerative diseases by inhibiting RIPK1 (17).

Furthermore, RIPK1 appears to play a significant role in promoting neuroinflammation in AD. Activation of RIPK1 mediates the transcriptional induction of Cst7, a biomarker of disease-associated microglia found near Aβ plaques in postmortem brain samples from AD patients and in AD mouse models (71). Studies demonstrated that treating an APP/PS1 Aβ-driven AD mouse model with Nec-1 effectively mitigates neuroinflammation and cognitive deficits. These findings suggest that RIPK1 might regulate neuroinflammatory pathways implicated in various neurodegenerative diseases and aging.

### 5.6 RIPK1 and autoimmune disease

Necroptosis plays a crucial role in the pathogenesis of numerous autoimmune diseases. Consequently, inhibiting RIPK1, a central regulator of necroptosis, emerges as a potential therapeutic approach. Genome-wide association studies (GWAS) have pinpointed around 100 non-HLA loci tied to rheumatoid arthritis (RA). This includes TNF, wherein specific SNPs and reduced levels of TNFAIP3 correlate with RA (72), GNE684, a RIPK1 inhibitor, has demonstrated promise, ameliorating pathological manifestations in a rodent collagen-induced arthritis model (73).

Necroptosis has also been connected to NETosis, a unique form of neutrophil cell death that results in the release of neutrophil extracellular traps (NETs). These NETs are involved in complement activation, leading to endothelial damage and triggering autoimmune vasculitis (71). Notably, RIPK1’s activation is essential for neutrophil NETosis. Inhibiting RIPK1 has shown efficacy in models of NET-driven diseases, including autoimmune vasculitis, venous thrombosis, and Systemic Lupus Erythematosus (SLE) (74).

Both the classical and alternative complement pathways have significance in autoimmune diseases such as SLE. When these pathways are activated under disease conditions, complement-dependent cytotoxicity ensues. Activation of the complement system can initiate RIPK1-RIPK3-MLKL-dependent necroptosis. Therefore, RIPK1 inhibitors might mitigate the harmful outcomes of complement-dependent cytotoxicity (75). These observations underscore RIPK1’s potential involvement in the development of neutrophil- and complement-driven autoimmune diseases.

### 5.7 RIPK1 and traumatology

Polykratis et al. conducted an experiment where they introduced a mutation, changing the conserved aspartate (D) at position 138 to asparagine (D138N), creating Ripk1 D138N/D138N knock-in mice. These mice were then infected with the cowpox virus. Observations in TNF-treated wild-type cells revealed RIPK1 autophosphorylation, which was inhibited by the RIPK1 inhibitor, Nec-1 (76). This evidence suggests that either genetic or pharmacological inhibition of RIPK1 can prevent TNF-induced sepsis.

In an ischemia-reperfusion stroke model, excessive activation of RIPK1 results in the necroptosis of neurons and endothelial cells. By examining the roles of RIPK1 and RIPK3 signaling pathways in a mouse model of collagenase-induced cerebral hemorrhage (ICH), it was found that Nec-1 reduced the interaction between RIPK1 and RIPK3, and decreased propidium iodide (PI)-positive cell death. Moreover, Nec-1 inhibited the activation of microglia and the expression of proinflammatory genes, including tumor necrosis factor-a (TNF-α) and interleukin-1β (IL-1β), following ICH (77). Several studies corroborate the protective effects of RIPK1 inhibition after acute brain injury. However, transitioning RIPK1 inhibitors into clinical trials for such diseases presents significant challenges, mainly due to the complexities involved in acute brain injury clinical trials (78). Importantly, other ischemic injuries might benefit from the inhibition of RIPK1-induced necroptosis. For instance, utilizing RIPK1 inhibitors during ischemia-reperfusion injuries in organ transplantation might enhance allograft survival.

## 6 Recent R&D progress of RIPK1-related drugs

To better understand the role RIPK1 plays in disease and its potential therapeutic applications, we examined the emerging drugs targeting this molecule. RIPK1 possesses a distinct hydrophobic pocket that can allosterically regulate its activity (34, 35). All identified RIPK1 inhibitors to date act by binding to this pocket. Notably, the chemotypes of RIPK1 inhibitors include indole-hydantoins (e.g., Nec-1) (10, 34), benzoxazepinones (e.g., eclitasertib) (79), benzoxazepinones (e.g., eclitasertib) (80), among others (81).

**Figure 4:**
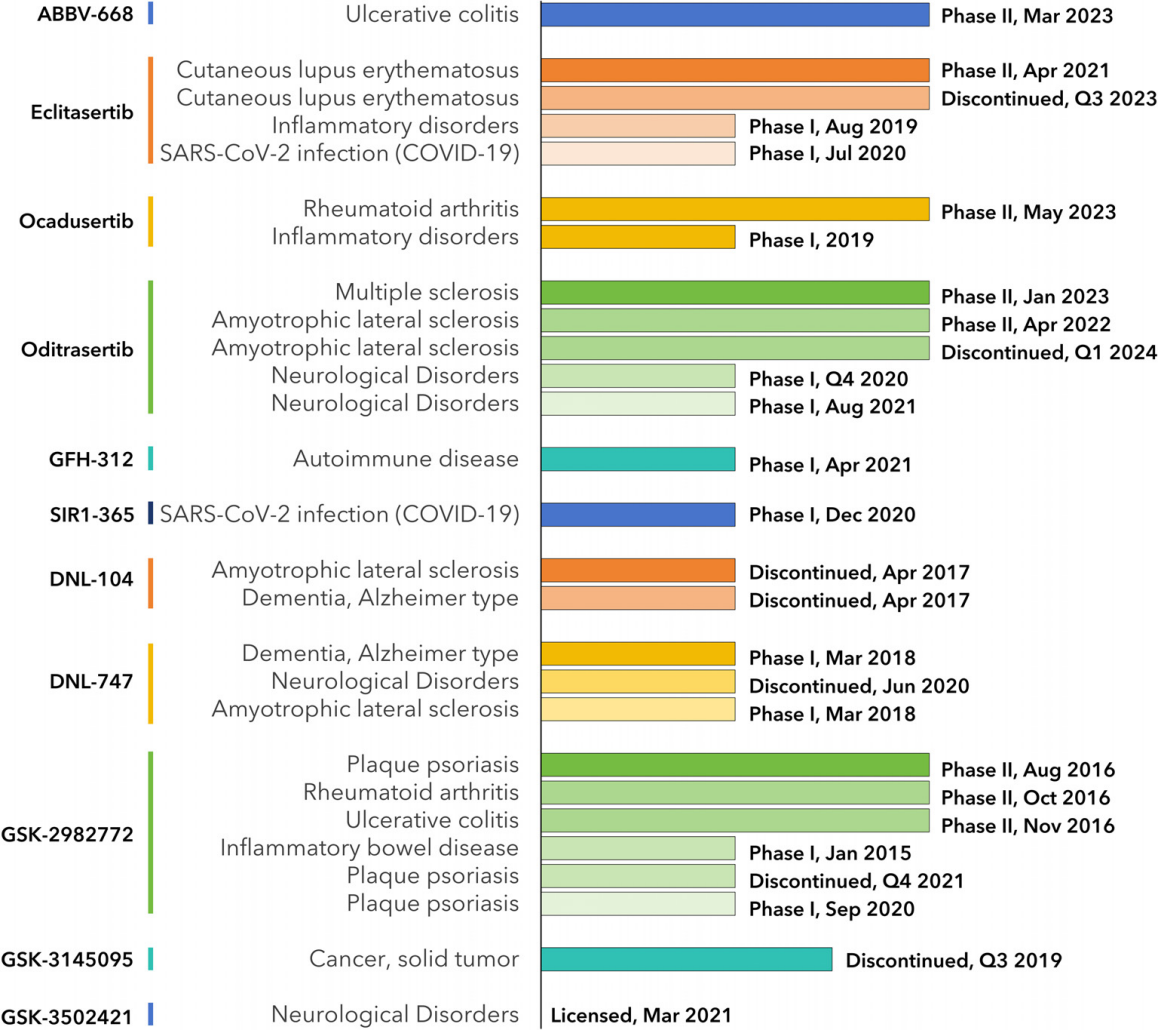
Milestones of RIPK1-inhibiting drug development. Data from CDDI database and is listed in **Supplementary Table S1**.

**Figure 5:**
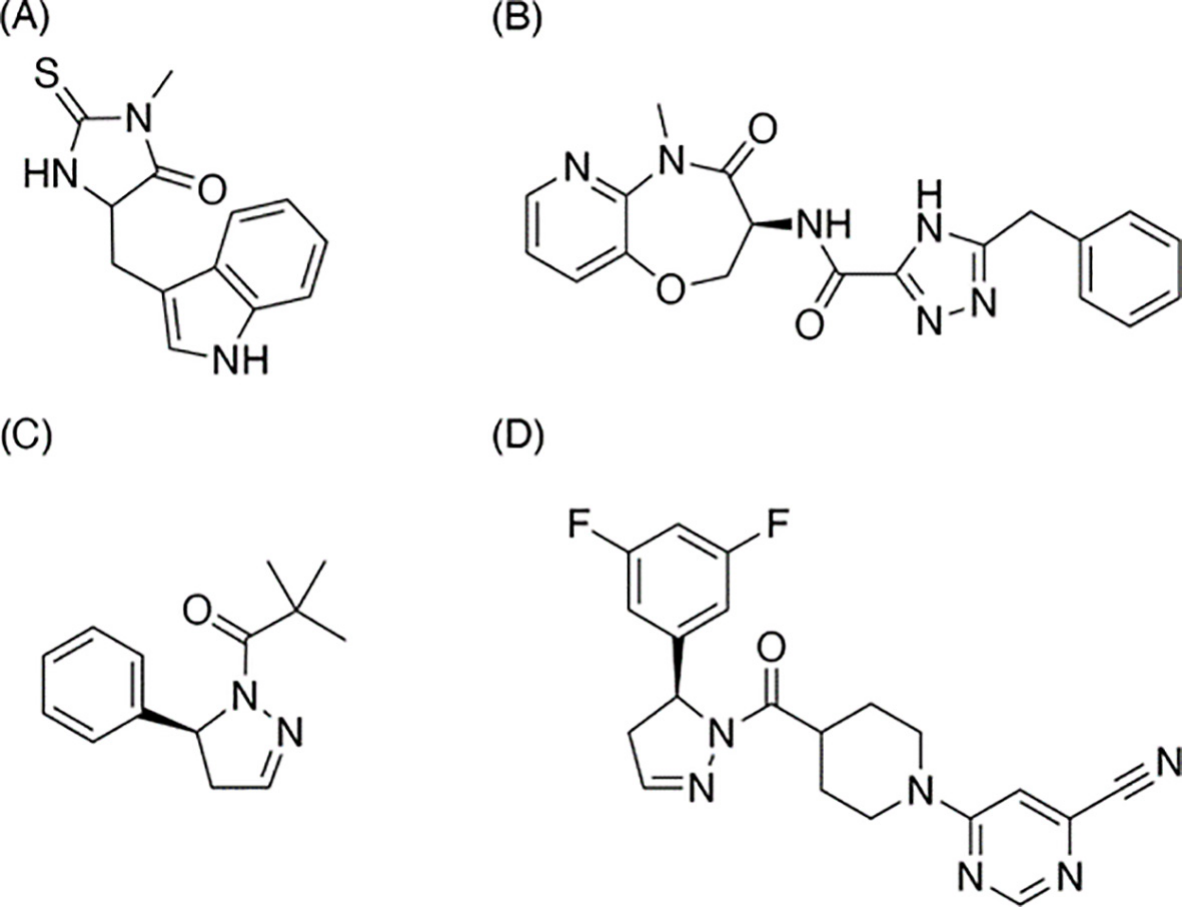
Structures of RIPK1 inhibitors mentioned in the article. **(A)** Necrostatin-1, the earliest-discovered and the most typical RIPK1 inhibitor and the earliest-discovered and the most typical necroptosis inhibitor; **(B)** Eclitasertib, the most progressed RIPK1-relevant drug under active development; **(C)** GSK-963, an inhibitor discontinued research as a drug; **(D)** RIP1i, the analog of GSK-963, shows a feature of better oral exposure and better medicinal potential.

In our search within the CDDI database, we identified 774 terms of RIPK1-associated drugs. From this set, 344 possess known pharmacological properties, 14 have a documented development status, 10 are actively under development, and none are currently on the market. Utilizing available public data, we collated these findings in **Supplementary Table S1**. In the following sections, we will delve deeper into a select group of these drugs (See **Figures 4**, **5**).

### 6.1 Necrostatin-1

Introduced and developed by Degterev et al. at Harvard Medical School (**Figure 5A**), Nec-1 stands out as both the inaugural RIPK1 inhibitor and the archetype for necroptosis inhibitors (10, 34). The discovery of Nec-1’s inhibitory effects significantly influenced the definition of necroptosis. This inhibitor dimerizes the RIPK1 kinase domain in a dose-dependent fashion, effectively curtailing the activity of overexpressed RIPK1. Parallel to deciphering Nec-1’s mechanism, the team also explored its structure-activity relationship (SAR). While certain alkylated analogs were ineffective, modifications like substituting oxygen for sulfur and introducing a chlorine atom on the indole ring retained the inhibitor’s potency. Interestingly, small-group substitutions on the indole ring enhanced Nec-1’s inhibitory capabilities. Although Nec-1 achieved the preclinical phase, its further development as a drug was halted. Instead, it transitioned into a “tool compound,” serving as a control in various drug preclinical studies (81).

### 6.2 Eclitasertib

Also known by its codes, DNL-758 or SAR-443122, eclitasertib (**Figure 5B**) has emerged as the most advanced RIPK1-targeting drug currently in development. Originating from Denali and developed in collaboration with Sanofi, this benzoxazepinone-chemotype RIPK1 inhibitor offers potential anti-inflammatory and immunomodulatory properties (82, 83). Regrettably, comprehensive studies detailing its exact mechanism remain unpublished. At present, Sanofi is conducting Phase 2 clinical trials to explore its efficacy in treating conditions like cutaneous lupus erythematosus (84), amyotrophic lateral sclerosis (85), and ulcerative colitis (86).

### 6.3 RIP1i

Developed by Wang et al. in collaboration with GlaxoSmithKline (GSK) (87), RIP1i (**Figure 5D**) binds to the allosteric pocket of RIPK1, situated between its N-terminal and C-terminal domains, adjacent to the ATP binding site. In comparison to its analog, GSK-963 (**Figure 5C**), RIP1i exhibits superior oral bioavailability, rendering it a promising candidate for medicinal applications. Over a 6-week oral regimen in mice, RIP1i maintained a consistently high serum concentration—a feat that Nec-1 couldn’t replicate. Additionally, RIP1i showcased promising *in vivo* tolerance without noticeable pathological implications.

Subsequent research proposed that RIPK1 kinase might facilitate macrophage-mediated adaptive immune tolerance in pancreatic cancer. However, this assertion was later contested (73). In 2019, a joint effort by Harris et al. and GSK ventured into a combination therapy involving RIP1i and a checkpoint inhibitor for pancreatic ductal adenocarcinoma (PDA). Though this research reached the investigational new drug (IND) phase, making it eligible for clinical trials, it was eventually abandoned in late 2019. Despite this setback, the insights gleaned from this study might lay the groundwork for devising new combination therapies centered around RIPK1.

### 6.4 Zharp1-211

**Figure 6:**
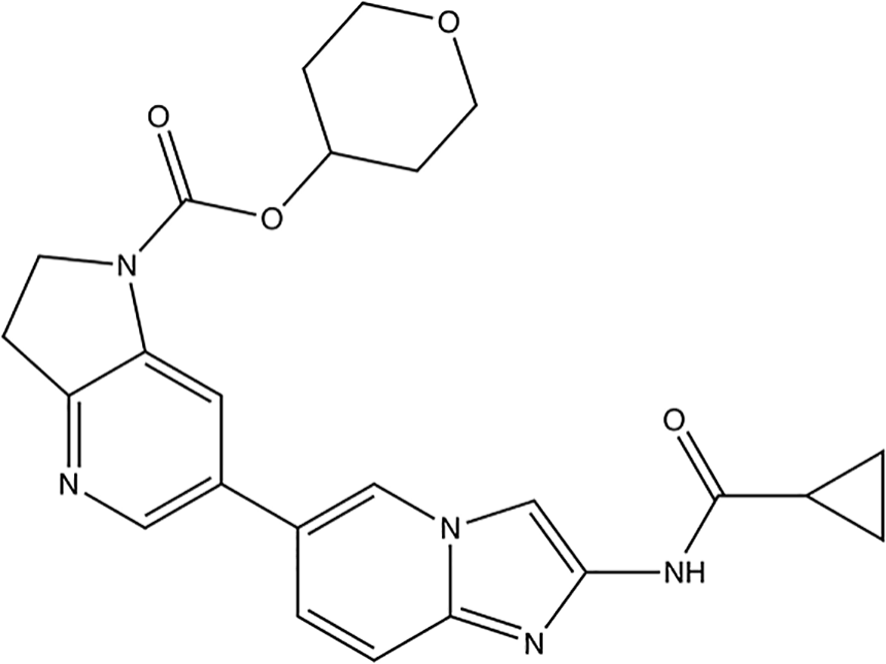
Structure of the new RIPK1 inhibitor Zharp1-211.

Active researches on potential RIPK1 inhibitors are still ongoing. The most recent report highlights Zharp1-211 (**Figure 6**), a novel RIPK1 inhibitor discovered by Yu et al. which plays a role in reducing graft-versus-host disease, or GVHD (88).

RIPK1/RIPK3 in IECs is required to trigger local and systemic GVHD via a mechanism involving both cell death and activation of a necroptosis-independent inflammatory cascade. RIPK1/RIPK3 promotes JAK/STAT1-induced production of chemokines and MHC class II molecules in IECs, which amplify the recruitment and expansion of alloreactive T cells. It is showed that alloantigen-primed T-cell–derived IFN-γ activates the RIPK1/RIPK3/JAK1/STAT1 axis in IECs to trigger a feed-forward inflammatory cascade, thereby amplifying alloreactive T-cell responses in the gastrointestinal (GI) tract and inducing subsequent conversion to systemic GVHD syndrome.

Zharp1-211 is an analog of the author group’s previous product, PK68 (89), and is considered the most potent inhibitor among which. Series of experiments including selectivity analysis and molecular docking shows that Zharp1-211 is an RIPK1 kinase inhibitor targeting the adenosine triphosphate-binding pocket in RIPK1’s inactive DLG (Asp156 (D)–Leu157 (L)–Gly158 (G))-out kinase conformation (88). *In vitro* experiments shows that Zharp1-211 was highly potent for blocking TNF-induced necroptosis in human colon cancer HT-29 cells and mouse fibroblast L929 cells, and reduced IFN-γ–induced STAT1 activation in mouse intestinal crypt cells by effecting JAK1/STAT1 pathway. Application in mice indicate that Zharp1-211 inhibition of RIPK1 strongly reduces an inflammatory cascade in the GI tract and inhibits of GVHD.

After allo-HCT, donor immune cells have the ability to eliminate host leukemic cells, which is known as the GVL effect and is critical for preventing cancer relapse. Authors finally evaluated the impact of Zharp1-211 on the GVL effect in B6 recipients receiving acute promyelocytic leukemia (APL) cells, which are driven by the PML-RARA fusion gene and recapitulate the human disease.

## 7 Conclusion and perspectives

RIPK1 stands at the crossroads of the necroptosis pathway, wielding a profound influence over its regulation. By modulating RIPK1’s activity, one can sway the balance of necroptosis, a process implicated in an array of organ and tissue injuries, cancers, and immunodeficiencies. Notably, the N-terminal kinase domain of RIPK1 is pivotal in instigating cell death, positioning it as a prime target for therapeutic interventions.

As of now, a great varieties of drugs centered around RIPK1 modulation is either already researched or under fervent development. Examples span across various chemotypes: indole-hydantoins (e.g., Nec-1), benzoxazepinones (e.g., eclitasertib), and dihydropyrazoles (e.g., RIP1i). Some RIPK1 inhibitors, like DNL747 and DNL748, has shown their potentials in Alzheimer’s and amyotrophic lateral sclerosis (90). Yet, their journey through clinical trials remains ongoing, paving the way for potential commercial availability in the future.

This research discusses mainly in tissues which expresses RIPK1 significantly, limiting us from discussing some diseases closely relevant to necroptosis but not RIPK1, such as oral squamous cell carcinoma (91) and breast cancer (92).

In the grand tapestry of biomedical research, our understanding of necroptosis and RIPK1 is continually evolving. However, a clear link between necroptosis and disease remains elusive, in large part because there are no clinically relevant methods to detect this cell death pathway in tissues (93). As more insights emerge about their intricate structures, mechanisms, and the dynamics of their inhibitors, optimism swells about the imminent clinical applications. In this realm, the future for RIPK1, necroptosis, and their therapeutic potential appears promising and luminous. Revealing necropoptotic signaling in more detail may answer these questions and provide a deeper understanding of RIPK1 in necropoptosis and its role in human disease.
